# Significant random signatures reveals new biomarker for breast cancer

**DOI:** 10.1186/s12920-019-0609-1

**Published:** 2019-11-08

**Authors:** Elnaz Saberi Ansar, Changiz Eslahchii, Mahsa Rahimi, Lobat Geranpayeh, Marzieh Ebrahimi, Rosa Aghdam, Gwenneg Kerdivel

**Affiliations:** 1 Curie Institute, INSERM U830, Translational Research Department, PSL Research University, Paris, 75005 France; 2 Department of Computer Sciences, Faculty of Mathematical Sciences, Shahid-Beheshti University, GC, Tehran, Iran; 3 School of Biological Sciences, Institute for Research in Fundamental Sciences (IPM), Tehran, Iran; 4 Department of Stem Cells and Developmental Biology, Cell Science Research Center, Royan Institute for Stem Cell Biology and Technology, ACECR, Tehran, Iran; 5 Department of Surgery, Sina Hospital, Tehran University of Medical Sciences, Tehran, Iran; 6 Institut Cochin, Department Development, Reproduction, Inserm U1016, CNRS, UMR 8104, Université Paris Descartes UMR-S1016, Paris, 75014 France

**Keywords:** Random signature, Network diffusion, Biomarker, Breast cancer, TAT (Tyrosine Aminotransferase)

## Abstract

**Background:**

In 2012, Venet et al. proposed that at least in the case of breast cancer, most published signatures are not significantly more associated with outcome than randomly generated signatures. They suggested that nominal *p*-value is not a good estimator to show the significance of a signature. Therefore, one can reasonably postulate that some information might be present in such significant random signatures.

**Methods:**

In this research, first we show that, using an empirical *p*-value, these published signatures are more significant than their nominal *p*-values. In other words, the proposed empirical *p*-value can be considered as a complimentary criterion for nominal *p*-value to distinguish random signatures from significant ones. Secondly, we develop a novel computational method to extract information that are embedded within significant random signatures. In our method, a score is assigned to each gene based on the number of times it appears in significant random signatures. Then, these scores are diffused through a protein-protein interaction network and a permutation procedure is used to determine the genes with significant scores. The genes with significant scores are considered as the set of significant genes.

**Results:**

First, we applied our method on the breast cancer dataset NKI to achieve a set of significant genes in breast cancer considering significant random signatures. Secondly, prognostic performance of the computed set of significant genes is evaluated using DMFS and RFS datasets. We have observed that the top ranked genes from this set can successfully separate patients with poor prognosis from those with good prognosis. Finally, we investigated the expression pattern of TAT, the first gene reported in our set, in malignant breast cancer vs. adjacent normal tissue and mammospheres.

**Conclusion:**

Applying the method, we found a set of significant genes in breast cancer, including TAT, a gene that has never been reported as an important gene in breast cancer. Our results show that the expression of TAT is repressed in tumors suggesting that this gene could act as a tumor suppressor in breast cancer and could be used as a new biomarker.

## Background

Cancer is a complex disease caused by uncontrolled division of abnormal cells in the body. This uncontrolled division is usually due to one or several mutations on so-called cancer driver genes, that will increase survival and proliferation of the cells under the good microenvironmental conditions. Breast cancer is a major leading cause of death among women [1]. Some evidence show that a rare population of the cells inside tumor are responsible for growth, development, invasion and metastasis [2, 3]. Therefore, discovering and controlling the mechanisms that regulate self-renewal and metastasis in tumors before they reach the late stage is essential for personalized patient care [4, 5]. Different cancer driver genes have been described in breast cancer, including TP53, BRCA1 and PALB2 [6]. Cancer genes do not act separately and deregulation of various genes from different pathways can lead to cancer initiation or progression [7, 8]. These genes give selective advantages to the cells, leading to profound changes in the cellular and also molecular phenotype of the cancer cells as compare to their normal counterparts. Many transcriptomic studies have shown that cancer cells exhibit specific expression profiles and these profiles can be used to separate normal from cancer cells but also to classify tumor samples with different clinico-pathological features [9]. Classical methods aiming to find cancer driver genes by looking to mutations can failed to discover important prognostic or therapeutic targets that exhibit differential expression but without carrying mutations. For this reason substantial efforts have been made to predict gene signatures related to human cancer [10–17] and also cancer stem cells. Some methods are based on considering single gene features while others taking into account the functional relationships between genes by considering a predefined biological network such as a co-expression network [12, 16] or a protein–protein interaction (PPI) network [15, 17].

Recent studies report that the performance of many network-based methods is comparable to methods based on single genes, and they have limited improvement in gene signature stability over different datasets [12, 13]. However, some approaches that produce informative genes or sub-networks by considering functionally related genes have more success in overcoming this problem [14, 15]. An important task is the evaluation of the significance of a cancer signature. On the other hand, it is possible that many of the randomly created gene signature groups, similar to already known or predicted groups, be able to separate normal from cancer cells. This is very complicated to interpret the effectiveness of random genes in classifying samples. Many kinds of possibility should be checked before we set up a general finding about why these randomly selected genes contain the differential information in controls and diseases and generic causal disease genes are very important for discovering the true signatures.

Statistical tests are usually applied to identify the association between a signature and outcome [18–20]. In 2011, Venet et al. [21] reported that gene signatures unrelated to cancer are significantly associated with breast cancer outcome. They compared 48 published breast cancer outcome signatures to random signatures of identical size and showed that the generated random signatures could separate good and poor patients significantly, even with nominal *p*-values less than the nominal *p*-values of published signatures. They suggested that nominal *p*-value is not a good estimator to show the significance of a signature and further hypothesized that such significant random signatures contain genes associated with proliferation and to a lesser extent cell cycle. In this research, we show that by using an empirical *p*-value, the published cancer-related signatures are more significant than random signatures and most of the random signatures are not significant with respect to empirical *p*-value. We show that random signatures with significant both nominal and empirical *p*-value are informative and can be used to predict genes that are highly associated to cancer (in our case breast cancer). To identify information in such random signatures, we introduce a novel method. Briefly, a score is assigned to each gene representing the frequency of its presence in the significant random signatures. The scores are then diffused through a PPI network and a permutation procedure is used to determine the genes with significant scores. The subset of genes whose scores are significant is considered as the set of significant genes. This computational methodology is applied to NKI cohort [10] that is a breast cancer dataset studied by Venet et al. to compute a set of significant genes. The disease association of this set is investigated using the GAD tool in David Functional Annotation server [22]. It is shown that this set is significantly related to breast cancer. To evaluate the prognostic performance of the computed set of significant genes, we use Distant Metastasis-Fee Survival (DMFS) and Recurrence-Free Survival (RFS) datasets [12] organized by Amsterdam Classification Evaluation Suite (ACES) by compiling a large cohort of breast cancer samples from the National Center for Biotechnology Information’s (NCBI’s) Gene Expression Omnibus (GEO). The results show that the top ranked genes from the set of significant genes set can successfully separate patients with poor and good prognosis in these datasets. To further investigate the function of the set of significant genes, pathway enrichment analysis is performed. Interestingly, the enriched significant pathways are highly related to cancer specially breast cancer and can separate patients with poor prognosis from those with good prognosis. Finally, we investigated the association of the top 10 genes with breast cancer. Among them, only Tyrosine aminotransferase (TAT) which is the first rank genes is not reported as a significant gene in cancer and we showed that this gene is frequently down regulated in tumor samples of breast cancer. Therefore, we suggest TAT as a novel biomarker in breast cancer tumor and its potential as tumor-suppressor gene should be further investigated.

## Methods

### Computing the empirical *p*-value for a signature

To compute the nominal *p*-value for a signature (or random signature), similar to Venet et al. [21], the 295 patients of the NKI cohort [10] and the overall survival end-points are considered and the same outcome association estimation procedure is used. First, the cohort is split based on the median of the first principal component (PC1) of a signature. Then, given this binary stratification of the cohort, the (observed) nominal *p*-value of this signature is computed using the standard Cox procedure (R package) [23]. Then the empirical *p*-value is computed based on permutation procedure [14]. Permutation test is a statistical tool for constructing sampling distributions. Similar to bootstrapping, permutation test builds sampling distribution by resampling the observed data points. Under the null hypothesis in permutation test, the sample labels are exchangeable i.e. the outcome is independent from the observed variables [14, 24]. By permuting the outcome values during the test, we observe many possible alternative outcomes and evaluate the significance of the true labels using calculated nominal *p*-values. In NKI cohort, we randomly shuffle the labels (N or ∼*N*) and compare the nominal *p*-values for each of the 48 breast cancer signature groups to 1000 nominal *p*-value which are obtained by permutation process. For k-th breast cancer signature group with $p_{k}^{nominal}$ and 1000 nominal *p*-value *p*(1),*p*(2),...,*p*(1000) which are resulted by permutation process, the Benjamini-Hochberg (BH) procedure controls the False Discovery Rate (FDR) in multiple testing experiments [25]. Indeed, for a given *α* and ordered sequence of 1001 nominal *p*-values, the adjusted *p*-values based on BH methods are calculated as:

1 $$\begin{array}{@{}rcl@{}} p_{(i)}^{BH}=\min \left(p_{(i)}\frac{m}{i},p_{(i+1)}^{BH}\right). \end{array} $$

**Fig. 1:**
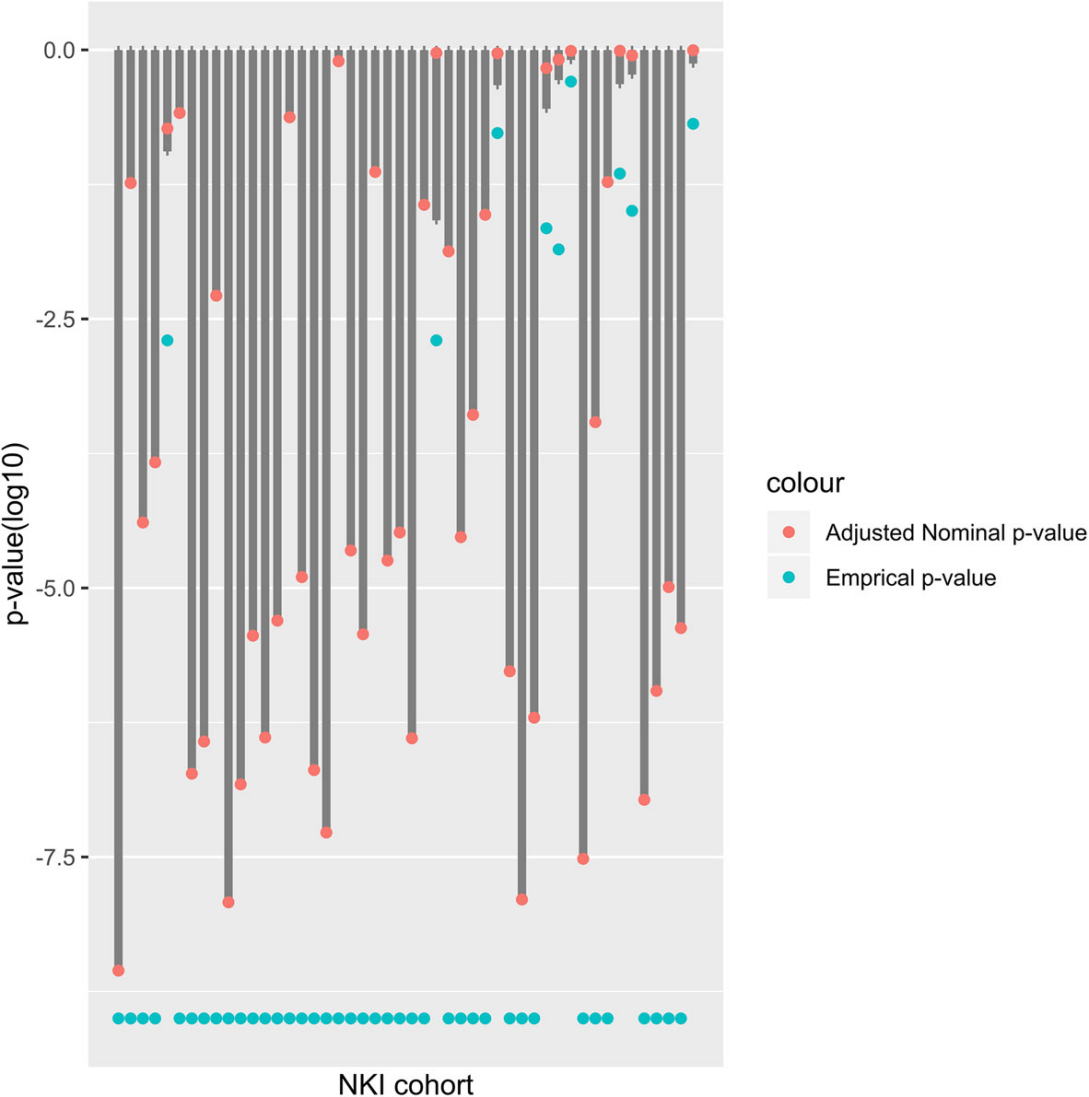
Adjusted nominal and range of adjusted nominal *p*-values related to 1000 permutation tests of the 48 breast cancer signatures. Red dots indicate adjusted nominal *p*-values and the grey lines are the range of adjusted nominal *p*-values from 1000 permutations. Blue dots show empirical *p*-value

For k-th breast cancer signature group, the *p*-value of the permutation test, called empirical *p*-value, is equal to the fraction of the 1000 adjusted nominal *p*-values that are equal or less than the adjusted nominal *p*-value of k-th group ($p_{k}^{BH}$), as shown in Eq. 2.

2 $$\begin{array}{@{}rcl@{}} p_{k}^{emprical}=\frac{\left|\left\{i|p_{(i)}^{BH} \leq p_{k}^{BH}\right\}\right|}{1000},~~~~~~~1\leq i \leq 1000, \end{array} $$

where $p_{(i)}^{BH} $ is the adjusted nominal *p*-value of *i*-th permutation test. The discoveries, i.e. the significant tests, are those with an empirical *p*-value less than *α*=0.05. The values of the adjusted nominal *p*-value and adjusted nominal *p*-values for 1000 permutations related to the 48 breast cancer signature groups are shown in Fig. 1.

The red dots indicate adjusted nominal *p*-values of 48 breast cancer signature groups and the grey lines are the range of adjusted nominal *p*-values for permutations. From this figure, we can see that the adjusted nominal *p*-values of the signatures are less than the adjusted nominal *p*-values of the permuted samples, which indicates the ability of empirical *p*-value in distinguishing normal and cancer groups. The blue dots show the empirical *p*-value of 48 breast cancer signature groups. In eight signatures out of 48, the adjusted nominal *p*-values are in the range of adjusted nominal *p*-values for 1000 permutation, so these eight signatures can not separate normal and cancer groups significantly.

### Meta-analysis and diffusion kernel approach to extract the information embedded in significant random signatures

In a complex disease like cancer, genes do not act in isolation and the interactions between them play a significant role [7, 8]. To take these interactions into account, the corresponding protein of each gene is considered and a PPI network is inferred using STRING database [26]. All the Entrez ID from the expression dataset and the Ensembl protein ID from STRING database are mapped to their gene name (HUGO symbol). The interactions between proteins in STRING database include physical and functional associations. In our algorithm, the evidence of conserved neighbors, co-occurrence, fusion co-expression and experiments are used to derive the interactions. Considering the significant random signatures, a score is assigned to each gene based on the number of times it is observed in these signatures. For example, a gene that occurs in 20 significant random signatures will get a score of 20. Let n be the number of genes and *S*=(*S*_1_,*S*_2_,...,*S*_*n*_) be the score of the genes. In this step, we construct a weighted graph G with nodes corresponding to the genes. Each node of G gets the score of its corresponding gene and the weights of the edges of G are the interaction scores between proteins coded by genes, which are obtained from STRING. The score of an interaction shows the confidence prediction of that interaction. The gene scores are diffused through G using the diffusion kernel of Kondor and Lafferty [15, 27], as described below:Laplacian matrix for simple graphs is defined as *H*=*D*−*A*, where *D* is the degree matrix and *A* is the graph’s adjacency matrix. For simple graph *G*, *A* is a zero-one matrix which all its diagonal entries are zero. Also, the *i*th diagonal entry of matrix *D* is the sum of the entries in the *i*th row of *A*. A similar approach can be used for constructing the laplacian matrix for weighted graph *G*. In this case, the *ij*th entry of the matrix *A* is the weight of the edge between the genes *i* and *j*. Similarly, the *i*th diagonal entry of matrix *D* will be the sum of the entries in *i*th row of *A*. In this case, the Laplacian matrix is also defined as *H*=*D*−*A*. Considering *w*_*ij*_ as the weight of the edge between genes *i* and *j* in graph G, the Laplacian matrix H for graph G is defined as *H*=[*H*_*ij*_], where:

3 $$ H_{ij}=\left\{ \begin{array}{ll} -W_{ij}& \quad if \ i\neq j\\ \sum_{l \neq i}W_{il} & \quad if \ i=j \end{array} \right.  $$

The diffusion kernel with generator H and bandwidth *β* is defined as:

4 $$\begin{array}{@{}rcl@{}} k_{\beta }=e^{\beta H}, \end{array} $$

where *β* shows the diffusion strength. For low diffusion strength kernels, scores are diffused only to a few well-connected neighbors but for high diffusion strength kernels, scores are diffused to distant nodes through the network. In this work, *β* is considered to be 0.3 since in [27] it is reported to achieve the least error rate in the breast cancer dataset. Using the matrix *k*_*β*_ the new scores, diffusion scores, for the genes are computed as follows:

5 $$\begin{array}{@{}rcl@{}} S_{\beta }=k_{\beta }.S. \end{array} $$

In fact, the diffusion score of one gene is based on its score, its neighbors scores and the score of its distant nodes.

### Identifying significant genes by permutation procedure

To determine the significance of diffusion scores of genes, the following random permutation procedure is used. Let *S*_*β*_=(*S*_*β*_(1),*S*_*β*_(2),…,*S*_*β*_(*n*)) where *S*_*β*_(*i*) denotes the diffusion score of gene *i* and *φ*_1_,*φ*_2_,...,*φ*_1000_ be 1000 random permutation on {1,2,..,*n*}. $S_{\beta }^{\varphi _{r}}=(S_{\beta }(\varphi _{r}(1)), S_{\beta }(\varphi _{r}(2)),\ldots,S_{\beta }(\varphi _{r}(n)))$ is constructed 1000 random permutation of *S*_*β*_ according to *φ*_1_,*φ*_2_,...,*φ*_1000_. We constructed 1000 random diffusion scores $ S_{\beta }^{r} $, as follows:

6 $$\begin{array}{@{}rcl@{}} S_{\beta }^{r}=k_{\beta }S_{\beta}^{\varphi_{r}}, ~~~~~~~~~~\text{for}~ 1\leq r\leq 1000. \end{array} $$

Let $ S_{\beta }^{r} \left (j \right) $ be the random diffusion score of gene j in vector $ S_{\beta }^{r} $. The null set $\{S_{\beta }^{r}(j)|1 \leq r \leq 1000 \}$ is considered for this gene. Then, the permutation score of *S*_*β*_(*j*) is computed by:

7 $$\begin{array}{@{}rcl@{}} \frac{|\{S_{\beta }^{r}(j) |S_{\beta }^{r} (j) \geq S_{\beta }(j)\}|}{1000}. \end{array} $$

The genes, which have permutation score less than 0.05 are considered as the set of significant genes. The set of significant gens are first sorted with respect to their permutation score and then based on their scores.

### Computing a pathway-score

Let SG be the set of significant genes computed by the method. For the pathway P, let the set *P*_*SG*_={*g*_1_,*g*_2_,...,*g*_*k*_} be the genes in SG which are presented in pathway P. Each gene *g*_*i*_ in P _SG_ is given two values, and is computed using the following equations:

8 $$\begin{array}{@{}rcl@{}} \mu_{N} \left(g_{i} \right) = \sum_{p_{j} \in N}^{}\frac{e_{g_{i},p_{j}}}{ \vert N \vert }, ~~~~~\mu_{\sim N} \left(g_{i} \right) = \sum_{p_{j} \in \sim N}^{}\frac{e_{g_{i},p_{j}}}{ \vert \sim N \vert}, \end{array} $$

where *p*_*j*_ ranges over the patients of phenotype *N* or ∼*N* and $ e_{g_{i},p_{j}} $ denotes the gene expression value of gene *g*_*i*_ in patient *p*_*j*_. Similar to the procedure mentioned in Lim et. al. [14], considering each patient *p*_*k*_ in phenotype ∼*N*, we define two new scores for pathway P:

9 $$\begin{array}{@{}rcl@{}} score_{N}^{p_{k}} \left(P \right) = \sum_{g_{i} \in P_{SG}}^{}e_{g_{i},p_{k}} \cdot \left(e_{g_{i},p_{k}}- \mu_{N} \left(g_{i} \right) \right)^{2}. \end{array} $$

10 $$\begin{array}{@{}rcl@{}} score_{\sim N}^{p_{k}} \left(P \right) = \sum_{g_{i \in P_{SG}}}^{}e_{g_{i},p_{k}} \cdot \left(e_{g_{i},p_{k}}- \mu_{\sim N} \left(g_{i} \right) \right)^{2}. \end{array} $$

$score_{N}^{p_{k}}(P)$ and $score_{\sim N}^{p_{k}}(P)$ are obtained based on a weighted mean approach. For instance, $score_{N}^{p_{k}}(P)$ is a weighted mean of values $(e_{g_{i},p_{k}}- \mu _{N})^{2}$, with corresponding non-negative weights as $e_{g_{i},p_{k}}$. In this formula, the weights are the gene expression values for genes in SG presented in pathway *P*. We use the non-negative terms $(e_{g_{i},p_{k}}- \mu _{N})^{2}$ and $(e_{g_{i},p_{k}}- \mu _{\sim {N}})^{2}$ as a measure of the difference in the gene expressions of normal and cancer groups, respectively.

### Patients and cell line selection

The ethics committee at the Royan Institute approved this study, and all the patients gave written informed consent on the use of clinical specimens for medical research. Ten breast cancer patients undergoing curative resection are included in this study. The median age of patients is 50 years (range 37-58 years). All patients are diagnosed with invasive ductal carcinoma; four of them are also metastatic. All patients underwent curative surgery, however three of them experienced neo-adjuvant therapy pre surgery. Both tumor and adjacent non-tumor tissue (the adjacent non-tumor tissue is defined as at least 1-cm distance from the tumor edge) are processed immediately after operation. The expression of TAT is evaluated by quantitative real-time polymerase chain reaction (RT-PCR) in all ten paired specimens. Among breast cancer cell lines MCF7 (is characterized as metastatic, ER+, PR+/-, HER2- and Luminal A type) and MDA-MB231 (is characterized as metastatic, ER-, PR-, HER2-, Claudin-low type and highly invasive) are selected and subjected to mammospheres formation and further analysis for TAT expression.

### RNA extraction and quantitative real-time polymerase chain reaction (qRT-PCR)

The expression of TAT (Tyrosine aminotransferase) is assessed by specific primer (F: 5’ATGCTGATCTCTGTTATGGG3’, R: 5’ CACATCGTTCTCAAATTCTGG3’) in tumor, normal and cell lines, respectively. Briefly, all specimens are preserved at -80 ^∘^C until RNA extraction. Total RNA is isolated using Trizol reagent (Qiagen, USA) and treated with DNAse I (Fermentas, USA) for 30 minutes in order to digest the genomic DNA. The quality of RNA samples is monitored by agarose gel electrophoresis and a spectrophotometer (Biowave II, UK). A total of 2 *μ*g of RNA is reverse transcribed with a cDNA synthesis kit (Fermentas, USA) and random hexamer primers according to the manufacturer’s instructions. Transcript levels are determined using the SYBR Green master mix (Takara, Japan) and a Rotorgene 6000. Expression of genes is normalized to the GAPDH housekeeping gene (F: 5’CTCATTTCCTGGTATGACAACGA3’, R: 5’CTTCCTCTTGTGCTCTTGCT3’). Relative quantification of gene expression is calculated using the △△Ct method.

### Monolayer and mammosphere culture

MCF-7, MDA-MB231 cell lines are purchased from Iranian Biological Resource Center, Tehran, Iran. The cell lines are cultured in DMEM–Dulbecco’s Modified Eagle Medium (GIBCO, USA) supplemented with 10% heat inactivated fetal bovine serum, (FBS; Invitrogen), 1% non-essential amino acid, 2 mM L-glutamine and 1% penicillin/streptomycin at 437 ^∘^C using a 5% CO2 standard cell culture incubator. For the mammospheres experiments, tissue culture plates are coated with poly hydroxyethyl methacrylate (pHEMA) to prevent cell attachment. Then 2 x 10e4 cells of each cell lines are cultured in poly hema coated flask and in serum-free medium consisted of DMEM medium supplemented with 20 ng/mL epidermal growth factor (Royan Institute, Iran), 20 ng/mL basic fibroblast growth factor (Royan Institute, Iran), 2% B27 (GIBCO, USA) and 2 mM L-Glutamine. All flask are incubated at 37 ^∘^C under a 5% humidified CO2 atmosphere for 10 days. Sphere structures are counted using an Olympus-IX71 fluorescent microscope. When the spheroids, reached to about 50 *μ*m diameter, are collected and pooled by gentle centrifugation, they are enzymatically dissociated with trypsin (GIBCO, USA) and subjected for RNA extraction.

### Statistical analysis

mRNA transcriptional levels in the tumor and matched non-tumor tissue are compared. Since the sample size is small (10 patients), we use the non-parametric Wilcoxon Rank Sum Test with the null hypothesis that both normal and cancer populations have same distributions. The alternative hypothesis is that the gene expression distribution for tumor group is shifted to the left. With Wilcoxon statistic as *W*=75, the resulted *p*-value is calculated as 0.03191, which rejects the *H*_0_ with *α*=0.05. For further validation, we also used bootstrap method for testing the differences in two populations. The test is repeated 1000 times and the *p*-value of Wilcoxon test are calculated. The median of the *p*-values of 1000 Wilcoxon test is calculated. The point estimate of the bootstrap method is 0.05158232, which is consistent with the results from Wilcoxon test.

## Results

### Computing empirical *p*-value for published breast cancer signatures

**Fig. 2:**
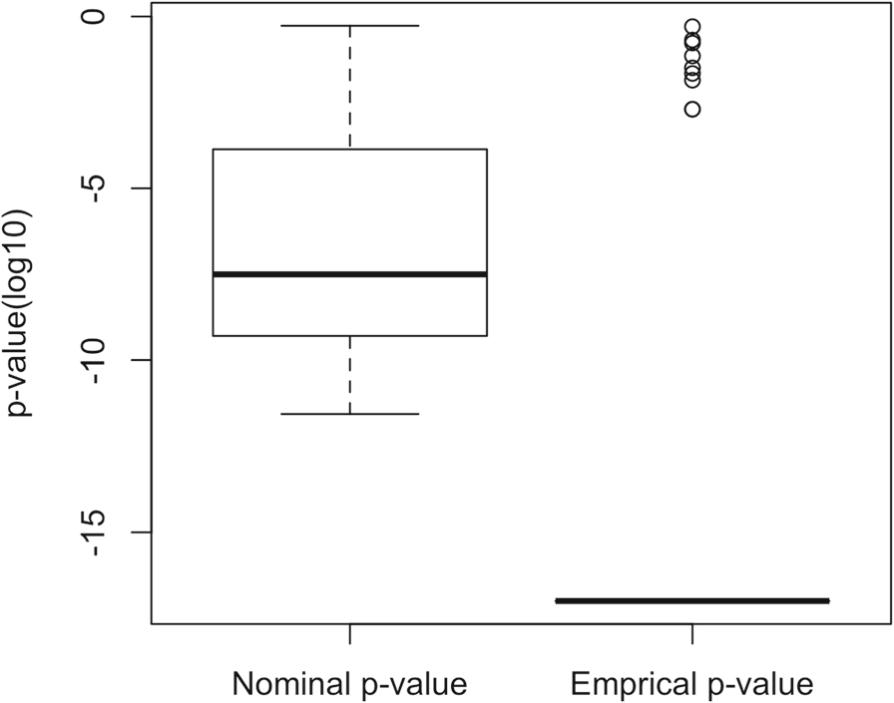
Nominal and empirical *p*-values of 48 published breast cancer signatures

In [21], Venet et al. showed from the 48 published breast cancer outcome signatures that statistically significant nominal *p*-values are not better than randomly generated signatures of identical size and hence the nominal *p*-values are not reliable. Thus, we use an empirical *p*-value (see “Methods”) to test the significance of nominal *p*-values by establishing whether the nominal *p*-value of a signature is lower than expected by chance. Figure 2 shows the nominal *p*-values of the 48 published breast cancer signatures and the empirical *p*-value achieved by permutation procedure (see “Methods”). The associated empirical *p*-values of the published breast cancer signatures are mostly less than 10^−15^. As depicted in this figure, the empirical *p*-values of the 48 published breast cancer signatures are mostly significant while the corresponding nominal *p*-values may not be significant.

### Extracting significant genes embedded in empirically significant random signatures

**Fig. 3:**
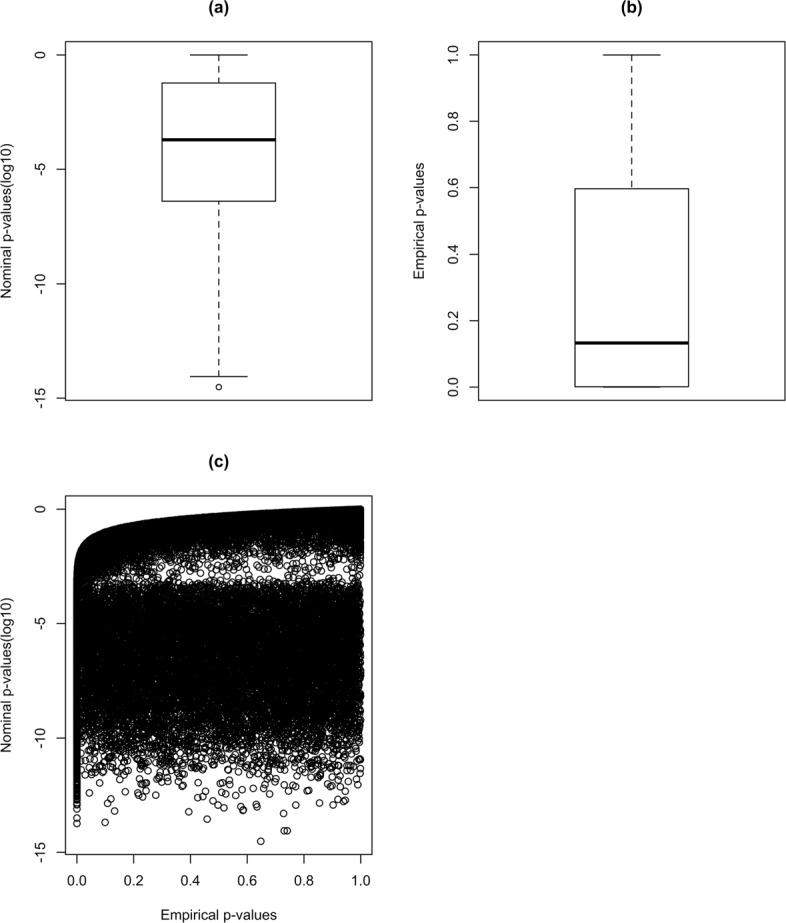
The boxplots and scatter plot for nominal and empirical *p*-values for the 48,000 random signatures. **a** The boxplot of nominal *p*-values, **b** the boxplot of empirical *p*-values. **c** The scatter plot of empirical *p*-value versus nominal *p*-value

Like Venet et al. [21], we also hypothesize that significant random signatures contain information. We introduce a novel method to extract the biologically relevant information in significant random signatures (see “Methods”). To achieve a set of significant genes in breast cancer considering significant random signatures, we use the NKI cohort, which is a breast cancer dataset studied by Venet et al. [21]. To this end, a set of 1000 random signatures of identical size is generated for each of the 48 published breast cancer signatures. The random signatures are considered significant if they are associated with breast cancer outcome with both nominal and empirical *p*-values. To demonstrate this, we consider one of the 48 signature groups with 106 genes as an example. Firstly, we select 106 random genes from the set of all human genes. We then repeat this process 1000 times and construct 1000 random signatures of identical size. By using the same procedure for each 48 group of signatures, we obtain 48,000 random signatures. Parts (a) and (b) of Fig. 3 show the boxplots of the nominal and empirical *p*-values resulted by 48,000 random signatures, respectively. The obtained nominal *p*-values, shown in part (a), support the results in Venet et.al. [21]. Part (c) contains the scatter plot of the 48,000 random signatures. Each dot in this figure shows the empirical *p*-value versus nominal *p*-value for one random signatures. For selecting the significant random signatures, we used the thresholds of 0 and -10 for empirical and log nominal *p*-values, respectively. Using the mentioned thresholds, 937 signatures are selected which is nearly two percent of all the signatures. By applying the method described in “Identifying significant genes by permutation procedure” subsection, we are able to obtain a set of 840 significant genes (See Additional file 1).

### Disease association of significant genes

To investigate the association of the top ranked genes with disease, the Genetic Association Database (GAD) tool in David Functional Annotation server [22] is used. GAD is an archive of published genetic association studies, which allows analysis of complex common human genetic disease [28]. The top-level disease and disease class assigned by GAD, given the 840 top ranked genes, is breast cancer and cancer with *p*-value= 0.0007 and *p*-value= 0.00098, respectively. Table 1 shows the enriched disease and disease class achieved from different set of genes. It can be seen from this table that the disease classes of the other sets of genes other than the first 840 top ranked ones is not related to cancer. This clearly highlights how our method can extract meaningful information from significant random signature.

**Table 1:** Enriched disease and disease class achieved from different set of genes by GAD

Genes	DISEASE	*p*-value	DISEASE-CLASS	*p*-value
1000 1st Genes	Breast Cancer	7.00E-04	CANCER	9.80E-05
1000 2nd Genes	Oral Premalignant Lesions	5.10E-03	DEVELOPMENTAL	1.20E-01
1000 3rd Genes	Neural Tube Defects	2.50E-02	REPRODUCTION	2.50E-01
1000 6th Genes	Bone density; Pregnancy loss	6.90E-03	AGING	1.50E-01
1000 9th Genes	Height	2.50E-03	NORMAL VARIATION	7.00E-03
1000 12th Genes	Inflammatory Bowel Disease	2.30E-05	CHEMDEPENDENCY	7.50E-05

### Association of top 20 genes with DMFS and RFS datasets

To further investigate the importance of genes extracted with our method, the prognostic performance of the top significant genes is computed using DMFS and RFS datasets. These two data sets, introduced by Staiger et al. [12], are two cohorts of breast cancer samples in NCBIs GEO.

**Fig. 4:**
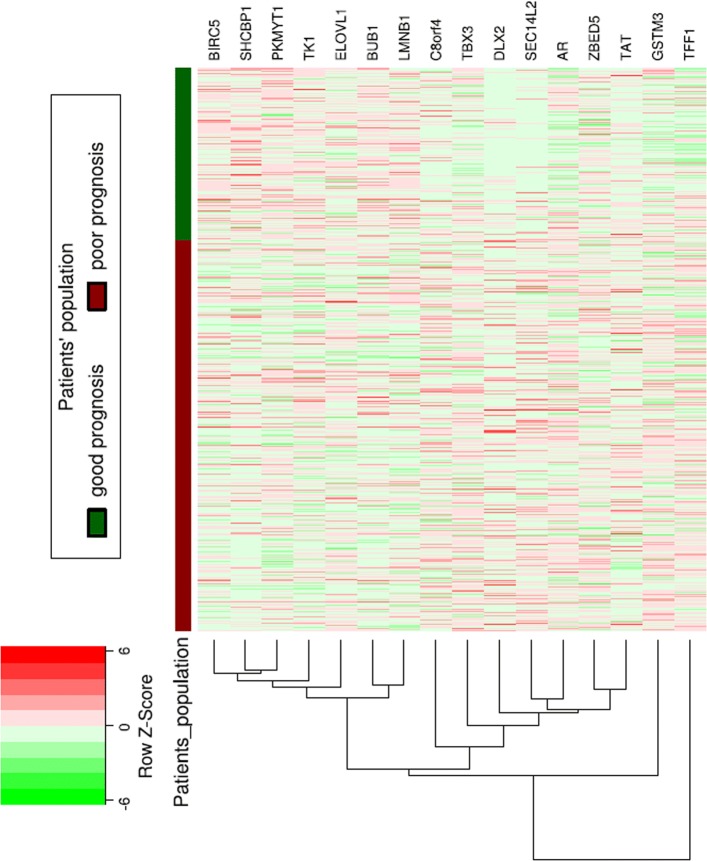
Expression of sixteen top-ranked genes in DMFS dataset

DMFS dataset is collected from six studies (Ivshina, Hatzis-Pusztai, Desmedt-June07, Miller, Schmidt, Loi) with 190 and 433 samples for poor and good prognosis, respectively. The RFS dataset contains 12 studies (Ivshina, Hatzis-Pusztai, Desmedt-June07, Minn, Miller, WangY-ErasmusMC, Schmidt, Pawitan, Symmans, Loi, Zhang, WangY) with 455 and 1161 samples for poor and good prognosis, respectively. The DMFS data set is a subset of the RFS data set. Their difference, however, is that in RFS data set, the patients are labeled according to recurrence-free survival whereas in DMFS data set, they are labeled according to distant metastasis-free survival. Among the top twenty significant genes computed previously, sixteen genes have gene expression information for studies in both DMFS and RFS datasets and 4 genes are eliminated in these studies since the gene expression values are not recorded for them. Expression of these sixteen genes for DMFS dataset is shown in Fig. 4. In both DMFS and RFS datasets, gene expression data for all studies are considered. Therefore, large number of samples with continuous gene expression values are available for analysis. By using t-test method, we confirmed that these genes can significantly separate the poor prognosis from good prognosis samples in DMFS and RFS datasets with *p*-values of 0.0017 and 0.0019, respectively.

### Prognosis value of the pathways associated with significant genes

To investigate the functions of the set of significant genes, hereinafter referred to as SG, pathways enrichment analysis is performed using ConsensusPathDB [29]. Only the pathways enriched with *p*-value less than 10^−9^ are considered (Table 2). Table 2 shows 22 enriched pathways from KEGG, Wikipathways, SMPDB and PID databases. Association of these pathways with cancer is surveyed through an extensive literature search. Among the 22 founded pathways, 14 of them are directly involved in cancer development and mostly contributed to cell cycle, proliferation and self-renewal ability. However, the remaining pathways indirectly affect tumor progression. The significance of these pathways is then evaluated using the DMFS and RFS datasets. To find the prognosis value of suggested pathways, a defined pathway-score is assigned to each patient and a statistical test is applied to distinguish the population of scores for phenotype *N* (good) and ∼*N* (poor). Considering pathway P, for each patient *p*_*k*_ in phenotype *N*, two scores, $score_{N}^{pk}(P)$ and $score_{\sim N}^{pk}(P)$, are defined (see “Methods” for more details). The population of pathway-scores, $score_{N}^{pk}(P)$ and $score_{\sim N}^{pk}(P)$, are supposed to vary for a pathway P that performs differently between the two phenotypes *N* and ∼*N*. Statistical t-test is applied for testing *H*_0_ (there is no important difference between pathway-scores) versus *H*_1_ (there is difference between pathway-scores). Most of the selected pathways can significantly separate the poor and good samples with significant *p*-values *p*−*value*<*α* (*α*=0.05).

**Table 2:** Enriched pathways using ConsensusPathDB

Pathway Name	Pathway Source	Pathway Size	Number of Enriched Genes	*p*-value in DMFS Dataset	*p*-value in RFS Dataset
Oocyte meiosis - Homo sapiens	KEGG	113	30	0.008	0.005
HTLV-I infection - Homo sapiens	KEGG	259	32	0.012	0.006
FoxO signaling pathway - Homo sapiens	KEGG	134	13	0.075	0.040
Cell cycle - Homo sapiens	KEGG	124	51	0.008	0.007
MAPK signaling pathway - Homo sapiens	KEGG	257	11	0.020	0.062
p53 signaling pathway - Homo sapiens	KEGG	68	13	0.010	0.004
Pathways in cancer - Homo sapiens	KEGG	398	32	0.319	0.463
DNA replication - Homo sapiens	KEGG	36	19	0.130	0.087
miR-targeted genes in lymphocytes - TarBase	Wikipathways		31	0.019	0.071
miR-targeted genes in epithelium - TarBase	Wikipathways	327	25	0.003	0.068
Gastric cancer network 2	Wikipathways	32	9	0.021	0.014
Mitotic G2-G2-M phases	Wikipathways	5	5	0.002	0.001
DNA Damage Response	Wikipathways	68	21	0.025	0.015
Cell Cycle	Wikipathways	103	39	0.029	0.051
Gastric Cancer Network 1	Wikipathways	29	10	0.010	0.007
Pyrimidine Metabolism	SMPDB	23	6	0.049	0.015
Validated targets of C-MYC transcriptional activation	PID	89	12	0.129	0.044
FOXM1 transcription factor network	PID	42	13	0.004	0.002
E2F transcription factor network	PID	75	23	0.051	0.029
Aurora B signaling	PID	41	18	0.013	0.012
Aurora A signaling	PID	31	8	0.003	0.015
PLK1 signaling events	PID	44	20	0.010	0.011

### Association of top 10 genes with cancer

To get a better insight in the importance of the significant genes extracted from empirically significant random signature, we investigated the role of the 10 most significant genes. Through extensive literature search, it is shown that most of the top 10 genes are reported to be associated with breast cancer or cancer in general. Table 3 presents a summary about the function of these genes. Among the listed genes, BIRC5, SEC14L2, Thymidine kinase (TK1), ZNF385B, CLIC6, ELOVL1, CHAF1B and TFF1 have been reported to have a role in early detection of cancers, tumor progression and metastasis in most of cancer types including breast cancer (see Table 3). PHYHD1 [30] is recently identified as a predictor for progression-free survival and metastasis in prostate cancers. Surprisingly, the most significant gene, TAT (Tyrosine aminotransferase), has not been reported to have a role in breast cancer. TAT encodes a mitochondrial enzyme mainly expressed in liver and contributes to metabolism and carbon metabolism pathways [31]. TAT gene is located on the chromosome 16 at position q22.2. Intriguingly, this chromosome is frequently deleted in many tumors including breast, liver, lung and gastric, suggesting the existence of a tumor suppressor gene within this region [31, 32]. Tumor suppressive mechanism of TAT gene has been previously reported in hepatocellular carcinomas (HCC). Indeed, down regulation of TAT is widely detected in primary HCC, which is significantly associated with either the loss of TAT allele or hyper methylation of TAT [32]. Induction of TAT into HCC cells prevents their tumorigenicity. Also, it has pro-apoptotic effect through the mitochondrial pathway [31]. Loss of chromosome 16q is widely reported in low tumor grade and luminal (ER+) breast cancer [31–35]. However, this study is the first one to suggest a role for this gene in breast cancer.

**Table 3:** Enriched pathways using ConsensusPathDB

Gene Name	Main functions	Included related pathway	Cancer type	Citations
TAT	Transaminase involved in tyrosine breakdown. Converts tyrosine to p-hydroxyphenylpyruvate. Pro-apoptotic effect through the mitochondrial pathway	Metabolism and carbon metabolism pathways in Mitochondria	Hepatocellular carcinomas (HCC), small cell carcinoma	[41]
BIRC5	Dual roles in promoting cell proliferation and preventing apoptosis. Essential for chromosome alignment and segregation during mitosis and cytokinesis. Participates in the organization of the center spindle by associating with polymerized microtubules.	Apoptosis, cell cycle, Immune system modulation	Breast, prostate, bladder, lung, colorectal, ovarian, cervical cancer and others	[42]
PHYHD1	Alpha-ketoglutarate-dependent dioxygenase activity	Peroxisomal phytanic acid alpha-oxidation pathway	Prostate cancer	[43]
SEC14L2	Carrier protein. May have a transcriptional activator activity via its association with alpha-tocopherol. May regulate cholesterol biosynthesis.	Transcription	Breast and prostate cancer	[44–46]
TK1	Catalyzes the addition of a gamma-phosphate group to thymidine. Biosyntehsis of dTTP, required for DNA replication.	Cell Cycle, Mitotic and Metabolism	Breast and prostate cancer	[30]
ZNF385B	Role in p53/TP53-mediated apoptosis.	Apoptotis	Breast and ovarian cancer	[35]
CLIC6	May insert into membranes and form chloride ion channels. May play a critical role in water-secreting cells, possibly through the regulation of chloride ion transport	Activation of cAMP-Dependent PKA, Hepatic ABC Transporters	Breast cancer	[47]
TCIM	Involved in the regulation of cell growth and differentiation. Involved in the regulation of heat shock response. Plays a role in the regulation of hematopoiesis even if the mechanisms are unknown (By similarity).	Apoptosis	Thyroid, breast, gastric, liver and lung cancer	[43, 48, 49]
ELOVL1	Fatty acids elongation	Metabolism and Regulation of lipid metabolism	Cancers	[31]
TFF1	Stabilizer of the mucous gel overlying the gastrointestinal mucosa that provides a physical barrier against various noxious agents. May inhibit the growth of calcium oxalate crystals in urine.	Estrogen signaling pathway, adhesion	Breast and gastric cancer	[50, 51]

### Expression pattern of TAT in malignant breast cancer vs. adjacent normal tissue and mammospheres vs. parental adherent cells

**Fig. 5:**
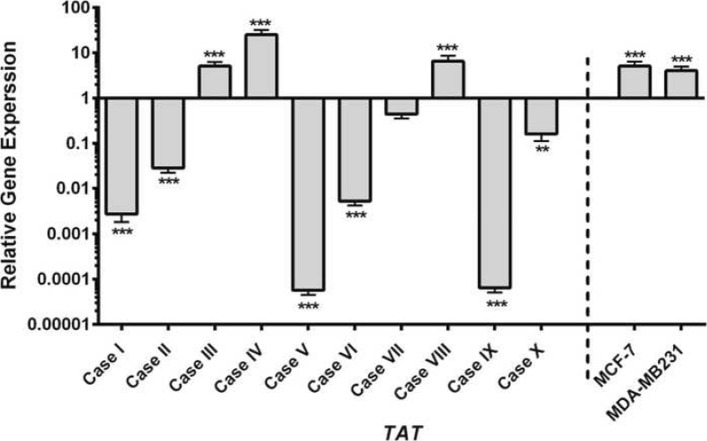
The expression of TAT gene in tumor vs. normal and spheres vs. parental cells. Left) Ten breast cancer patients enrolled in the present study and the expression pattern of TAT is evaluated using real time RT-PCR in tumoral and adjacent normal tissues. Seven of ten patients had down-regulation of TAT gene compared to normal tissues, but two of them over-expressed it. (Right) Both type of mammospheres derived from MCF-7 and MDA-MB-231 revealed enhanced expression of TAT. The bars in MCF-7 and MDA-MB-231 indicated the Mean ±SD of at least three different experiments. ***: *P*≤0.001

**Fig. 6:**
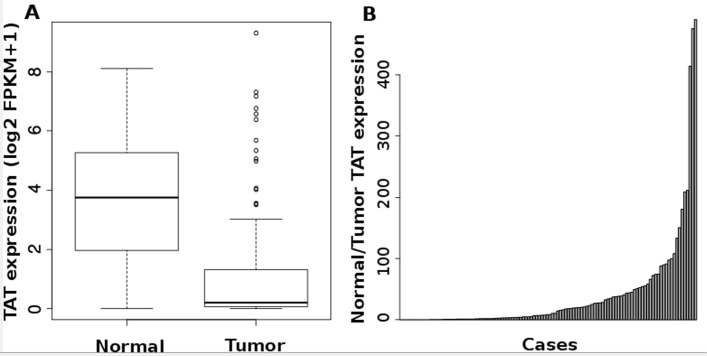
Expression of TAT in TCGA breast tumor and adjacent normal tissue. A. Primary tumor RNA-seq data and the associated normal tissue are available for 112 patients from the TCGA BRCA project. **a** Comparison of TAT expression (log2 FPKM+1) in tumors vs. adjacent normal tissue. Student t-test *p*-value is <10^−15^. **b** Ratio of TAT expression in normal tissue over expression in tumors for the 112 patients

Based on our data, we hypothesized that TAT could play an important role in breast cancer. Therefore, its expression is evaluated in breast tumor samples. All tumors in the present study are classified as invasive ductal carcinoma (IDC). Three samples are ER+, PR+ and HER2+. Three patients have undergone neoadjuvant therapy prior to surgery due to their histopathological characteristics and tumor stage. As shown in Fig. 5, in most of cases, TAT is under expressed as compared to adjacent normal tissue. However, two of them had over-expressed TAT genes. Surprisingly, the expression of TAT increased in mammospheres derived from MCF-7 and MDA-MB-231 as compared to their adherent counterparts (about 3.2 fold, *p*<0.001). The decreased expression of TAT in tumor as compared to normal tissue is confirmed in TCGA BRCA dataset. Only the cases for which both tumor and adjacent normal tissue RNA-seq data are available are considered for analysis. A massive and highly significant (*p*-value <10^−15^) decrease of TAT expression is observed in tumors as compared to their adjacent tissue in most of the samples (87/112, median decreased of 20 fold, Fig. 6).

## Discussion

Nominal *p*-values are most commonly used to show the significance of the observations. In 2012, Venet et.al. [21] suggested that nominal *p*-values are not reliable measures to show the significance of a human cancer signature and outcome. They showed that, at least in the case of breast cancer, signatures reported in the literature are no better than randomly generated signatures. To show this, they generated random signatures that could separate good and poor patients with significant nominal *p*-values. They further suggested that such significant random signatures are due to genes associated with proliferation and cell cycle.

In this research, we first show that by using the empirical *p*-values and considered it as a complimentary criterion for nominal *p*-value, most of the random signatures are not more significant than published signatures related to breast cancer. Next, we focused on that subset of random signatures with significant both empirical and nominal *p*-value. This subset of random signatures may contain some information that makes them be significant like published ones. To show that the significant random signatures are informative, we apply a computational method to extract information embedded within them. To do this, we define a novel scoring assignment method based on the number of the significant signatures that contain a specific gene to give a score to each gene. Since genes do not act in isolation in a complex disease like cancer and the interactions between them play a significant role, we consider the relationship of the genes in PPI network. To this end, a diffusion method on PPI network is used to smooth the score of the genes. Using a permutation method, the genes with significant score are selected as cancer-related genes.

We applied this method on the NKI cohort, which is a breast cancer dataset studied by Venet et al. [21] to achieve a set of significant genes in breast cancer. It is shown that this predicted set of genes is related to breast cancer. To evaluate the prognostic performance of the computed set of significant genes, we used two data sets of DMFS and RFS. They contain cohorts of 6 and 12 datasets from GEO, introduced by Staiger et al. [12]. We show that the set of significant genes can separate the poor and good prognosis in these datasets. To show the accuracy of this method, the following procedure is done. Firstly, pathways enrichment analysis using ConsensusPathDB is performed considering KEGG, Wikipathways, SMPDB and PID databases on this set of genes. All enriched pathways, including cell cycle, p53 signaling pathway and DNA Damage Response are associated with cancer development. Secondly, for most of the significant genes obtained by this method (all of the 10 most significant genes), a role in cancer initiation or progression has been described in multiple types of cancer. In fact, 8 out of these 10 genes have been shown or suspected to play key roles in breast cancer development (see Table 3), highlighting the effectiveness of our method. In addition, our method could effectively identify new important candidates for the cancer type being studied. It identified TAT which has not so far been reported in cancer. In summary, the obtained results demonstrate the accuracy of the proposed method as it can effectively extract meaningful information from a set of completely random signatures. This method allows the identification of genes with expressions that contain predictive values and are associated with cancer-related pathways. Finally, we checked the expression of TAT in human breast cancer tissues as well as mammospheres as a model of breast cancer stem cells. TAT is down regulated in most of the invasive ductal carcinoma patients (71%) used in this study and in TCGA patients from BRCA projects. Interestingly, a previous study reported that TAT, which is located on chromosome 16q, has a tumor suppressive role in hepatocellular carcinomas (HCC) [31]. Indeed, down regulation of TAT expression is widely detected in primary HCC, which is significantly associated with either the loss of TAT allele or hyper methylation of TAT. Induction of TAT into HCC cells prevents their tumorigenicity. TAT has been shown to exhibit pro-apoptotic effect through the mitochondrial pathway [31]. Although the role of TAT in breast cancer is unclear, the loss of chromosome 16q has been widely reported in low tumor grade and luminal (ER+) breast cancer [31–35]. The expression pattern of TAT is down regulated in seven of ten patients in the present study suggesting that loss or low expression of TAT could contribute to initiation or/and progression of breast cancer. However, TAT is up regulated in two patients as well as mammospheres derived from malignant breast cancer lines. Mammospheres is a model for enriching the breast cancer stem cells [36, 37]. There are several studies indicating that breast cancer stem cells are responsible to resistance to chemotherapy [38, 39] and induction of metastasis [40]. Therefore, the similarity of TAT expression in both mammospheres and the two of our patients can lead to the hypothesis that over expression of TAT may be associated with the resistance of tumor to therapy. This hypothesis can be the subject of study for future research.

## Conclusion

As a conclusion, random signatures can contain significant information to discover new cancer genes. The method we developed can be used to rank the genes extracted from significant random signatures and predict important signatures in cancer. In addition, this study is the first one to suggest a role of TAT in breast cancer. However, further investigations should be conducted to elucidate the putative tumor suppressor properties of TAT in breast cancer as well as its potential importance in stem cells, metastasis and resistance to drugs.

## Supplementary information

**Additional file 1** A set of 840 significant genes which is resulted by “Extracting significant genes embedded in empirically significant random signatures” subsection.
